# Draft genome sequence of ectomycorrhizal edible mushroom *Phlebopus portentosus* strain YAF023

**DOI:** 10.1128/mra.00588-24

**Published:** 2024-07-05

**Authors:** Mei Yang, Xiaolong Yuan, Lingjia Kong, Yuanni Gao, Chengyi Liu, Yi Wang

**Affiliations:** ^1^ Panzhihua Academy of Agricultural and Forestry Sciences, Panzhihua, Sichuan, China; 2 Laboratory of Forest Plant Cultivation and Utilization, Yunnan Academy of Forestry & Grassland, Kunming, Yunnan, China; University of California, Riverside, California, USA

**Keywords:** *Phlebopus portentosus*, genome, Illumina sequencing

## Abstract

*Phlebopus portentosus* is a favorite ectomycorrhizal edible mushroom in tropical China. Here, we present a draft genome sequence of *P. portentosus* strain YAF023. The genome resource will support subsequent research into the relationship between ectomycorrhizal fungi and trees.

## ANNOUNCEMENT

The genus *Phlebopus* (R. Heim) Singer, classified in the Boletinellaceae (Boletales), contains about 12 species and is widely distributed in tropical and subtropical areas (1). *Phlebopus portentosus* is a widely consumed ectomycorrhizal edible mushroom in China and Thailand (2). It is rich in nutrients, e.g., polysaccharides, amino acids, mineral elements, and pyrrole alkaloids (3). *P. portentosus* shows antimicrobial and cytotoxic properties (4, 5). *P. portentosus* is the first species of *Phlebopus* genus, which can be cultivated by providing carbon or nitrogen compounds in artificial media (6).

The ectomycorrhizal fungus *P. portentosus* strain YAF023 was isolated from the fruiting body grown in the underwood of *Pinus yunnanensis*. The isolation process was as follows. The fruiting body of *P. portentosus* was collected from Panzhihua, Sichuan Province, China (26°15′14″ N, 106°08′24″ E), in 2020. The fruiting body of *P. portentosus* was washed by soaking it in tap water for 2 hours, then rinsing it with sterile water. A tissue grinder was used to grind the fruiting body, after which sterile water was added to obtain a slurry. Finally, the fruiting body slurry was further diluted and spread onto a solid malt-yeast medium (Difco, Lawrence, KS, USA) containing 200 µg/mL of kanamycin and streptomycin, respectively. After 20 days of incubation (28°C), a single colony was transferred to a fresh malt-yeast medium. One single colony (strain YAF023) was identified by sequencing the internal transcribed spacer (ITS) region as belonging to *P. portentosus*, and the ITS sequence was deposited in GenBank (accession number: PP097691).

The mycelia of *P. portentosus* YAF023 were harvested for DNA extraction after cultivation in a malt-yeast liquid medium at 28°C for 25 days on a rotary shaker at 150 rpm. Genomic DNA was extracted using a DNeasy minikit (Qiagen, Valencia, CA, USA) following the manufacturer’s instructions. Illumina (San Diego, CA, USA) sequencing libraries were prepared using a TruSeq DNA Sample Prep Kit (Illumina) with insert sizes of 400 bp. The library was sequenced on an Illumina NovaSeq 6000 platform with a 2 × 150 bp paired-end configuration. A total of 30,594,648 raw reads were obtained (Table 1). High-quality cleaned sequences (4.62 Gb; coverage, 150×) were obtained by trimming the adapter sequences with AdapterRemoval version 2 (7). The *de novo* genome assembly was conducted using SOAP denovo version 2 (8). The default parameters were used for all the software unless otherwise specified. The genome assembly size of the YAF023 strain was 38,374,347 bp with 23 scaffolds. The longest scaffold was 4,540,672 bp, with an N50 value of 2,085,074 bp and an N90 value of 1,352,901 bp. The GC content was 48.00%. The completeness of the genome assembly was assessed using BUSCO version 5.2.2 (9) with the OrthoDBfungi v10 (fungi_odb10) database, and its completeness value was 95.90%.

**TABLE 1:** Genome sequencing statistics

Parameter	Value
Raw reads	
Read number	30,594,648
Total bases (bp)	4,619,791,848
Coverage	150×
Genome assembly	
Total base	38,374,347
Total scaffolds	23
Max sequence length	4,540,672 bp
N50	2,085,074 bp
N90	1,352,901 bp
GC_content (%)	48.00
Genome assembly assessment	
Complete BUSCOs	95.90%
Complete and single-copy BUSCOs	95.00%
Complete and duplicated BUSCOs	0.90%

## Data Availability

The draft genome sequence of *Phlebopus portentosus* YAF023 has been deposited at NCBI with accession number SAMN39487613, BioProject number PRJNA1066409, and SRA accession number SRR28483933 (GenBank accession number JAYXKI000000000) https://www.ncbi.nlm.nih.gov/datasets/genome/GCA_037892935.1/.
